# Combination of Neoadjuvant Gemcitabine‐Cisplatin and Anti‐Tuberculosis Therapy for a Patient With Muscle‐Invasive Bladder Cancer and Renal Granulomatosis That Progressed After Intravesical Bacillus Calmette‐Guérin Therapy

**DOI:** 10.1002/iju5.70057

**Published:** 2025-05-28

**Authors:** Takahiro Tsumori, Seiji Hoshi, Kei Yaginuma, Satoru Meguro, Kanako Matsuoka, Junya Hata, Yuichi Sato, Hidenori Akaihata, Soichiro Ogawa, Yoshiyuki Kojima

**Affiliations:** ^1^ Department of Urology Fukushima Medical University School of Medicine Fukushima Japan

**Keywords:** anti‐tuberculosis therapy, Bacillus Calmette‐Guérin, bladder cancer, neoadjuvant chemotherapy, renal granulomas

## Abstract

**Introduction:**

A case of muscle‐invasive bladder cancer and renal granulomatosis that developed after intravesical Bacillus Calmette‐Guérin therapy, in which a combination of neoadjuvant gemcitabine‐cisplatin and anti‐tuberculosis therapy was safely administered, and radical cystectomy was ultimately performed, is reported.

**Case Presentation:**

A 64‐year‐old man with non‐muscle‐invasive bladder cancer underwent transurethral resection and intravesical Bacillus Calmette‐Guérin therapy every time bladder cancer recurred. However, the patient developed left renal granulomatosis during treatment. Anti‐tuberculosis therapy was prioritized since there was no bladder cancer progression. However, local bladder cancer progression was observed during the anti‐tuberculosis therapy. To successfully cure the renal granulomatosis and suppress tumor progression, neoadjuvant gemcitabine‐cisplatin was combined with anti‐tuberculosis therapy for 2 months, followed by radical cystectomy. There were no gemcitabine‐cisplatin complications and no renal granulomatosis recurrence during combination therapy.

**Conclusion:**

Combination of gemcitabine‐cisplatin and anti‐tuberculosis therapy was possible for a patient with bladder cancer when Bacillus Calmette‐Guérin infection was under control.

**Summary**

- Renal granulomatosis is a rare complication of intravesical Bacillus Calmette‐Guérin therapy, and treatment strategies for patients with bladder cancer progression during treatment with anti‐tuberculosis therapy are unclear.
- We report a case of muscle‐invasive bladder cancer and renal granulomatosis that developed after intravesical Bacillus Calmette‐Guérin therapy, in which a combination of neoadjuvant gemcitabine‐cisplatin and anti‐tuberculosis therapy was safely administered.

**Abbreviations**
ATT: anti‐tuberculosis therapy
BCa: bladder cancer
BCG: Bacillus Calmette‐Guérin
CRP: C‐reactive protein
CT: computed tomography
GC: gemcitabine‐cisplatin
MRI: magnetic resonance imaging
RC: radical cystectomy

## 1 Introduction

Renal granulomatosis is a rare complication of intravesical Bacillus Calmette‐Guérin (BCG) therapy, and treatment strategies for patients with bladder cancer (BCa) progression during treatment with anti‐tuberculosis therapy (ATT) are unclear. A case of a patient with advanced BCa who safely received neoadjuvant gemcitabine‐cisplatin (GC) in combination with ATT for renal granulomatosis caused by BCG infection and underwent radical cystectomy (RC) successfully is reported.

## 2 Case Presentation

**FIGURE 1:**
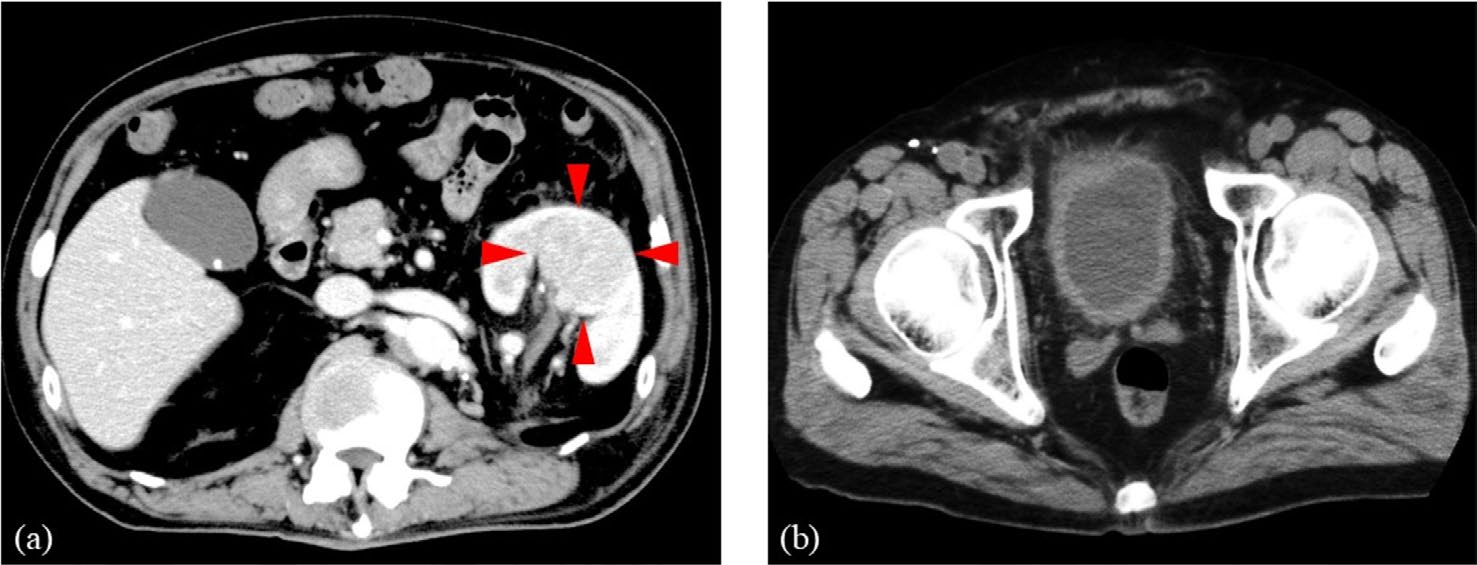
Contrast‐enhanced CT when the patient presented with persistent fever. (a) CT shows a 48 × 40 mm^2^ abscess in the left kidney. (b) CT shows no obvious bladder tumor.

**FIGURE 2:**
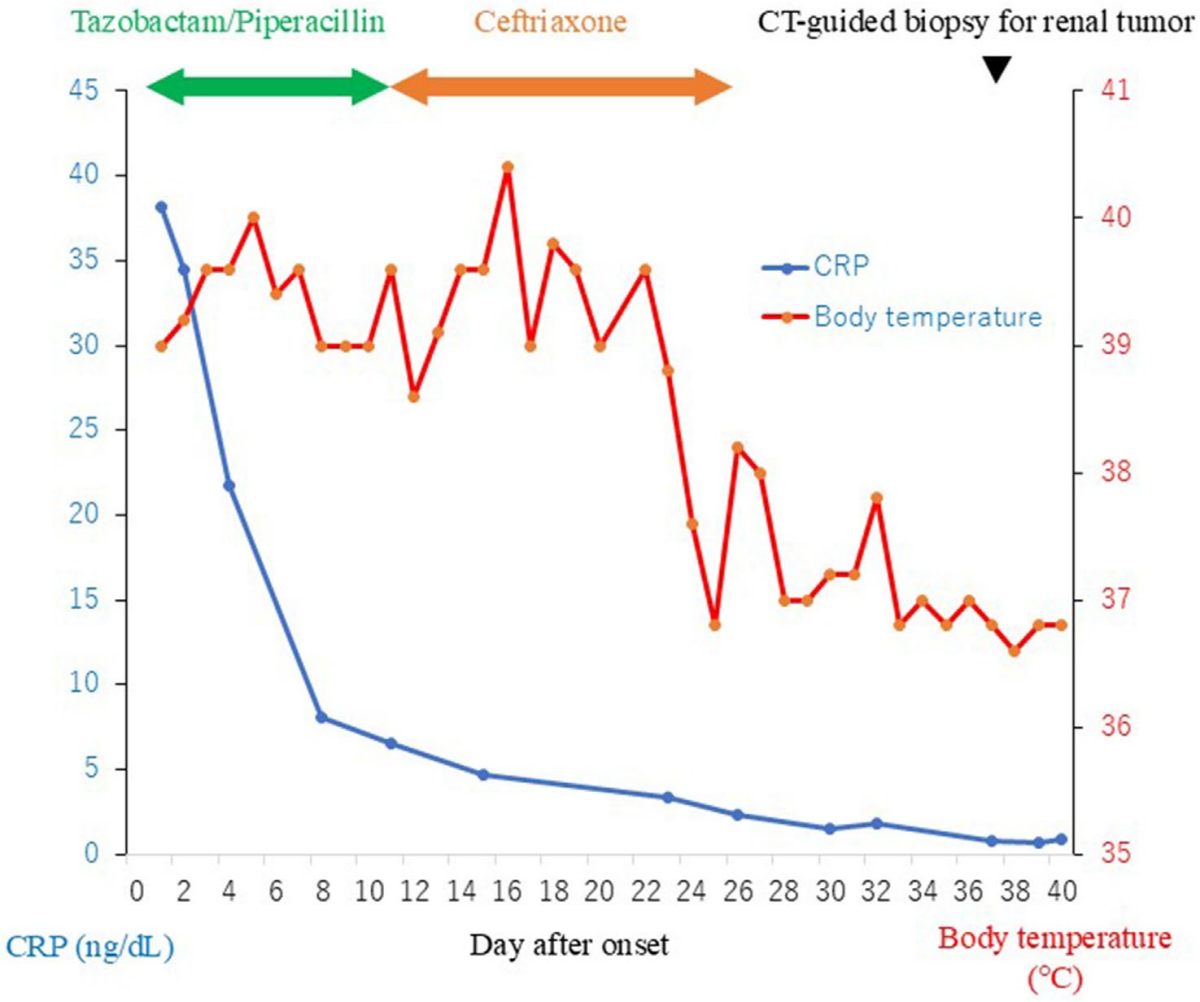
The course of treatment until CT‐guided biopsy for renal abscess. Although the CRP levels improved gradually after the start of antibiotic therapy, the fever persisted.

**FIGURE 3:**
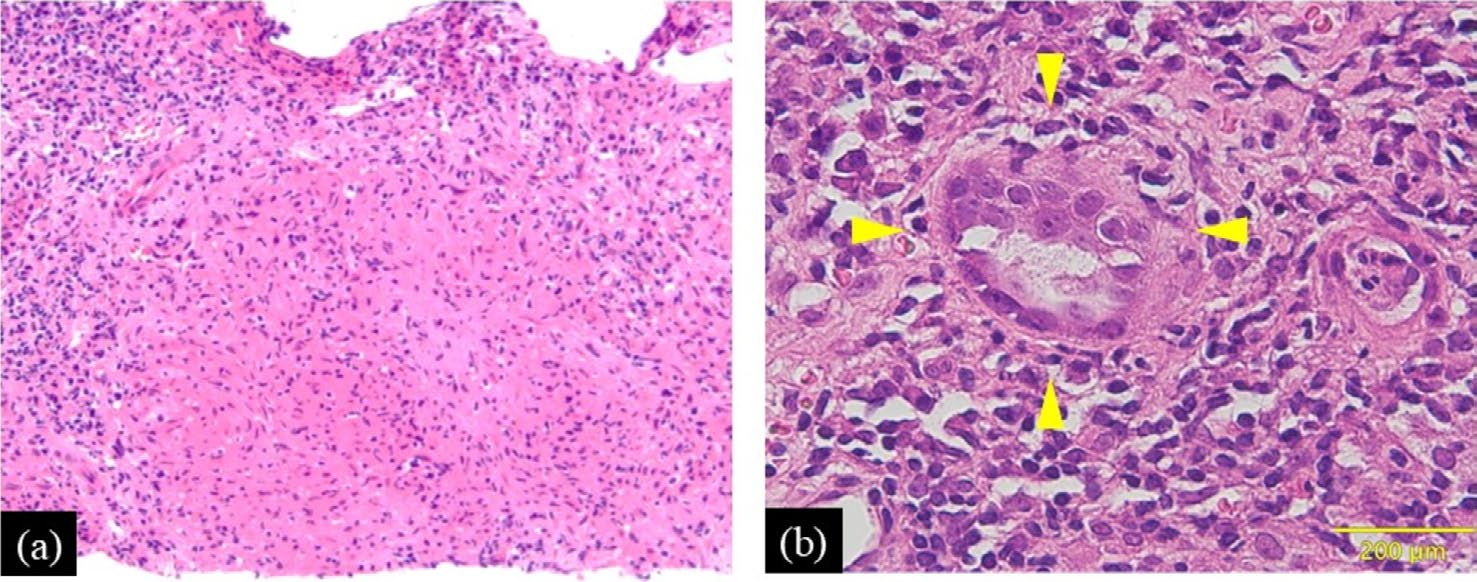
Hematoxylin and eosin staining of the renal abscess. Histological examination shows a granulomatous lesion (a) and Langhans giant cells (b).

A 64‐year‐old man with BCa (urothelial carcinoma, pT1, high grade) was started on postoperative intravesical BCG therapy in February 2020. The patient underwent six induction BCG therapy sessions, followed by maintenance therapy every three sessions. Intravesical BCG therapy was effective, although the BCa recurred three times when it was discontinued due to painful urination. We recommended RC, but the patient strongly preferred bladder preservation. Therefore, transurethral resection was performed in May 2022, and intravesical BCG therapy was subsequently resumed. However, during the therapy, the patient presented with persistent fever in July 2022. Laboratory examination showed significantly elevated C‐reactive protein (CRP) (38.2 mg/dL), but interferon‐γ release assay was negative. Computed tomography (CT) showed a 48 × 40 mm^2^ abscess in the left kidney (Figure 1a). There was no obvious bladder tumor on CT (Figure 1b), and the urinary cytology was negative, indicating no BCa recurrence. Antibiotic therapy (tazobactam/piperacillin and ceftriaxone) was initiated for a left renal abscess. Although the CRP levels improved gradually, the fever was prolonged (Figure 2). A positive urine mycobacterial culture suggested renal granulomatosis due to BCG infection. Consequently, CT‐guided biopsy of the renal abscess was performed. Histological examination with hematoxylin and eosin staining showed granulomatous lesions (Figure 3a) and Langhans giant cells (Figure 3b), which were diagnosed as renal granulomatosis caused by BCG infection.

**FIGURE 4:**
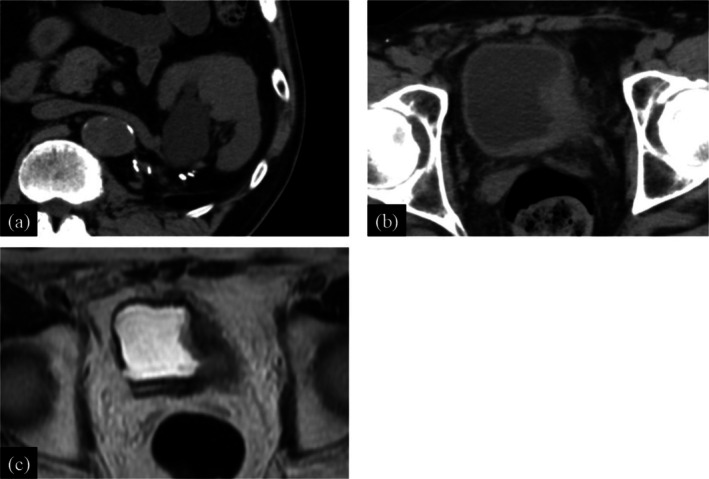
Plain CT images (a, b) and plain MRI image (c) 4 months after the start of anti‐tuberculosis therapy. (a) The size of the left kidney tumor has decreased, but hydronephrosis has appeared. (b) The size of the tumor on the left lateral wall of the bladder has increased. (c) The tumor on the left wall of the bladder shows extramural invasion.

A 6‐month ATT regimen (rifampicin 600 mg/day + isoniazid 300 mg/day + levofloxacin 500 mg/day) was planned. However, at the time of starting the therapy, urine cytology became positive, suggesting BCa recurrence, but CT showed no bladder tumor. ATT was prioritized with careful follow‐up for BCa, and RC was planned after the ATT was completed. ATT was initiated in December 2022 and continued until May 2023. Four months after starting ATT, CT showed a reduction in the size of the left renal abscess to 30 × 31 mm^2^ (Figure 4a), and urine mycobacterial culture turned negative. However, CT and magnetic resonance imaging showed BCa progression on the left lateral wall of the bladder without metastases (Figure 4b,c). There was concern about renal granulomatosis recurrence if ATT was interrupted to perform an immediate RC, due to the insufficient duration of treatment completed at that point. Therefore, from April 2023 to May 2023, two cycles of neoadjuvant GC (gemcitabine 1000 mg/m^2^ on days 1, 8, 15, and cisplatin 25 mg/m^2^ on day 2) were administered every 3 weeks combined with ATT for 2 months. During the GC, the patient experienced Grade 2 neutropenia and Grade 1 thrombocytopenia, as defined by the Common Terminology Criteria for Adverse Events version 5.0. Based on WHO criteria [1], the resolution of renal granulomatosis was confirmed by the completion of the planned ATT and the negativity of urine mycobacterial cultures, followed by robot‐assisted RC and ileal conduit construction in June 2023. Histopathological examination showed urothelial carcinoma, ypT3aN0, RM0, ly1, v1. This patient has continued to show no recurrence of renal granulomatosis, and there has been no BCa recurrence for 6 months.

## 3 Discussion

To the best of our knowledge, there have been no reports of chemotherapy, such as GC for BCa, while treating BCG infection, and its efficacy and safety have not been established. This is the first report in which the combination of neoadjuvant GC and ATT was safely administered, and RC was ultimately performed for the patient with muscle‐invasive BCa and renal granulomatosis that developed after intravesical BCG therapy.

Intravesical BCG therapy is an effective treatment for non‐muscle‐invasive BCa, but it is occasionally associated with serious complications such as renal infection [2, 3]. The incidence of renal infection after intravesical BCG therapy is 0.2%–2%, and renal granulomatosis occurred in < 0.1% of patients [4]. Therefore, the appropriate management of renal granulomatosis caused by BCG infection is unclear [5]. In general, for renal granulomatosis caused by BCG infection, 6 months of anti‐tuberculosis drugs were recommended in accordance with tuberculosis treatment, though the optimal duration of therapy has not been clarified [6], but shortening treatment duration may lead to renal granulomatosis recurrence [7]. In this case, since there was no evidence of BCa progression when renal granulomatosis developed, to cure the BCG infection, ATT was planned for a period of 6 months before performing the RC for BCG‐intolerant non‐muscle‐invasive BCa. However, 4 months into ATT, combining GC with ATT was considered necessary because the BCa had progressed. Although there are no reports of the safety of GC for BCa patients with renal granulomatosis caused by BCG infection, the safety and efficacy of combining chemotherapy and ATT have been reported in other cancers with tuberculosis [8, 9, 10]. When combining chemotherapy, neutropenia and thrombocytopenia, as well as liver dysfunction, are cited as adverse events associated with ATT. Although rifampicin induces CYP3A4 and CYP2C8, which may reduce the efficacy of some chemotherapies, this does not affect GC. In addition, because of the renal mass shrinkage that indicated improvement in renal granulomatosis, it was considered that the risk of renal granulomatosis recurrence became lower even if GC were introduced after 4 months of ATT.

Although the combination of GC and ATT was given safely in this case, it does not always apply to all patients. Because ATT generally requires 6 months, there is a risk of BCa progressing during treatment. Moreover, GC may cause immunosuppression [11], which increases the risk of BCG infection relapsing. Adverse events such as neutropenia and thrombocytopenia may necessitate a dose reduction of GC. Therefore, the combination therapy should be determined individually according to the symptoms of BCG infection and BCa progression. In this case, GC was safely administered 4 months after ATT when the BCG infection was under control. Regarding BCa, the present patient could wait to undergo RC because there was no obvious BCa progression at the start of ATT. This case suggests that the combination of GC and ATT may be safe after 4 months of ATT. Further research is needed to determine the efficacy and safety of the combination of GC and ATT.

## 4 Conclusion

This case, in which GC for BCa and ATT for renal granulomatosis caused by BCG infection were safely combined, suggests that this combination therapy may be safe when BCG infection is under control.

## Conflicts of Interest

The authors declare no conflicts of interest.
